# Rituximab in the Management of Autoimmune Bullous Diseases: A Treatment-Resistant Case Series from a Single Central European Referral Center

**DOI:** 10.3390/medicina60020270

**Published:** 2024-02-04

**Authors:** Maciej Marek Spałek, Magdalena Jałowska, Monika Bowszyc-Dmochowska, Marian Dmochowski

**Affiliations:** 1 Autoimmune Blistering Dermatoses Section, Department of Dermatology, Poznan University of Medical Sciences, 60-355 Poznan, Poland; mspalek@ump.edu.pl (M.M.S.); mjalowska@ump.edu.pl (M.J.); 2 Cutaneous Histopathology and Immunopathology Section, Department of Dermatology, Poznan University of Medical Sciences, 60-355 Poznan, Poland; m.bowdmo@wp.pl

**Keywords:** autoimmune bullous diseases, rituximab, desmoglein, glucocorticosteroids

## Abstract

*Background and Objectives*: Rituximab (RTX) has been the predominant treatment for autoimmune bullous diseases (AIBDs). The objective of this research was to assess the advantages and safety characteristics of RTX treatment in individuals with AIBD. This assessment focused on clinical remission and a reduction in glucocorticosteroid usage, its effect on the titers of autoantibodies targeting desmoglein-1 (DSG-1) and desmoglein-3 (DSG-3), and adverse occurrences during a 12-month follow-up period in a dermatology department within a Central European university context. *Materials and Methods*: Our case series involved eleven patients, including eight patients with pemphigus vulgaris, two with pemphigus foliaceus, and one with epidermolysis bullosa acquisita. They received a 1 g dose of rituximab, repeated over a two-week interval. *Results*: The reduction in a prednisone-equivalent dosage after 2, 6, and 12 months following the second RTX infusion was 65.05%, 73.99%, and 76.93%, in that order. The titers of antibodies against DSG-1 exhibited reductions of 43.29%, 75.86%, and 54.02% at 2, 6, and 12 months, respectively. By contrast, the antibody concentrations targeting DSG-3 displayed a decrease of 27.88%, 14.48%, and 5.09% at the corresponding time points. Over the course of the 12-month monitoring period, 18.18% of patients experienced disease relapse, while the remaining individuals achieved either complete or partial remission with minimal or no therapy. Adverse effects were noted in 36.36% of the patient population; they were mild, and no serious adverse effects were reported. *Conclusions*: RTX represents an efficacious and well-tolerated therapeutic option for the management of AIBD and merits consideration in cases of refractory AIBD. However, further research is imperative to delineate the most optimal dosage, dosing frequency, and total quantity of maintenance infusions required. Additionally, there is a compelling need for studies that explore the impact of RTX on individuals with AIBD who do not exhibit a significant reduction in anti-desmoglein autoantibody levels.

## 1. Introduction

Autoimmune bullous diseases (AIBDs) encompass a diverse range of dermatological conditions characterized by the immune system targeting specific skin structural proteins. They are diagnosed clinically through the observation of blisters and erosions on either the skin or mucous membranes [1]. Pemphigus represents a cluster of rare, persistent AIBD impacting the skin and/or mucous membranes. The hallmark feature of pemphigus is the immune system’s predominant attack on desmoglein (DSG) proteins, primarily DSG-3, DSG-1, or both DSG-3/DSG-1. However, it is important to note that other antibodies may also play a role in immune response [2,3].

Epidermolysis bullosa acquisita (EBA) pertains to the category of AIBD, representing a rare disease distinguished by a heterogeneous clinical manifestation and the occurrence of autoantibodies directed against collagen VII. Conventional therapeutic interventions involve the administration of glucocorticosteroids (GCS) and dapsone (DP); however, in instances of heightened severity, more sophisticated treatment modalities may be employed [4,5,6].

According to the treatment guidelines for pemphigus vulgaris (PV) and foliaceus (PF) formulated by the European Academy of Dermatology and Venereology in 2020, rituximab (RTX) was advocated as the primary therapeutic option irrespective of the specific type or severity of the bullous disease. Furthermore, in situations where RTX was not employed as the initial pharmaceutical intervention, guidelines suggested its inclusion as a secondary or tertiary line of treatment in conjunction with other therapeutic modalities [7].

Presently, there exists no established standard treatment protocol for EBA. Nevertheless, there have been reports in the literature pointing to the effectiveness of using RTX for managing this condition [8].

The primary objective of this investigation was to assess the efficacy of RTX in treating AIBD over a 12-month observational timeframe.

## 2. Materials and Methods

The retrospective analysis concentrated on reviewing the medical records of individuals diagnosed with AIBD who underwent RTX treatment at the dermatology department in Poznan from 2014 to 2022. The RTX treatment for severe immunosuppression-resistant pemphigus was funded through a national program supported by a government agency. The investigation applied the subsequent exclusion criteria:

- Patients with a prednisolone equivalent dosage below 10 mg/day before their initial RTX infusion, with the exclusion of those with absolute contraindications to GCS;
- Individuals presenting with less than 10 active lesions (encompassing blisters, erosions, or new areas of erythema) prior to their initial RTX infusion;
- Subjects lacking follow-up data for at least one year after completing the last RTX cycle;
- Individuals with a history of hypersensitivity reactions to the administered medication;
- Subjects with active and severe infections;
- Patients diagnosed with severe congestive heart failure (New York Heart Association Class IV).

Following the implementation of these exclusion criteria, 11 patients met the inclusion criteria and were included in the study.

The identification of PV, PF, and EBA involved the examination of clinical manifestations, histological results, and immunological investigations, including direct immunofluorescence (DIF), indirect immunofluorescence (IIF), and multiplex ELISA manufactured by Euroimmun (Luebeck, Germany) for the detection of IgG antibodies targeting DSG-1, DSG-3, BP 180, BP 230, envoplakin, and type VII collagen [9]. Sera were diluted in 1:101 PBS. ELISA levels were analyzed in 100 samples from healthy blood donors. The cut-off was set at 1.0, and the obtained prevalences were as follows: BP180—3.0%; BP230—1.0%; DSG-1—1.0%; DSG-3—1.0%; envoplakin—2.0%; and type VII collagen—0.0%.

All participants in the research received RTX treatment through intravenous infusions, with each infusion consisting of a 1 g dose, repeated at a 2-week interval. A total of 1 g per cycle was administered on a single day within a hospital setting. The ongoing surveillance of blood pressure and urine output occurred throughout every infusion. Before the initiation of each treatment cycle, thorough evaluations were undertaken, encompassing a complete blood count, assessments of renal and liver function, and a urine analysis. Follow-up evaluations were conducted at 2, 6, and 12 months after the completion of the last RTX course. Additionally, the ELISA for IgG antibodies was assessed before the initial RTX infusion and during subsequent follow-ups.

We adopted the criteria established by Murrell DF et al. for establishing the management of disease activity [10]. The complete remission of therapy (CRNT) was characterized by the non-existence of recent and/or pre-existing lesions while the patient refrained from treatment on a systemic level for a minimum of two months. If an individual remained lesion-free while on a prednisone dosage of 10 mg/day or below or with supplementary immunosuppressive agents, the activity of the disease was categorized as complete remission on minimal therapy (CRMT). Disease relapse (R) was characterized by the appearance of three or more new lesions within a month, which did not naturally resolve within one week. Partial remission on minimal therapy (PRMT) was defined by the occurrence of temporary new lesions that resolved within one week while the patient underwent minimal therapeutic interventions.

## 3. Results

Our case series study comprised a total of 11 participants (8 with PV, 2 with PF, and 1 with EBA). Among them were three males and eight females, with an average age of 52.73 ± 13.61 years (mean ± SD), ranging from 27 to 69 years. The duration between the diagnosis of the disease and the initiation of RTX therapy varied, ranging from a minimum of 1 year to a maximum of 12 years, with an average duration of 5.09 ± 3.78 years.

Comprehensive details regarding the general characteristics, treatment methods, and disease activity of the patients can be found in Table 1.

The mean Initial prednisone-equivalent dose stood at 30.56 ± 21.14 mg, which gradually tapered among individuals with managed or regulated disease. After the administration of the second RTX infusion, two months later, the mean prednisone equivalent dosage was 10.68 ± 10.34 mg. This figure further decreased to 7.95 ± 10.49 mg at 6 months and reached 7.05 ± 5.42 mg at the 12-month mark. Consequently, the reductions in dosage amounted to 65.05%, 73.99%, and 76.93% at intervals of 2, 6, and 12 months, in that order.

**Figure 1:**
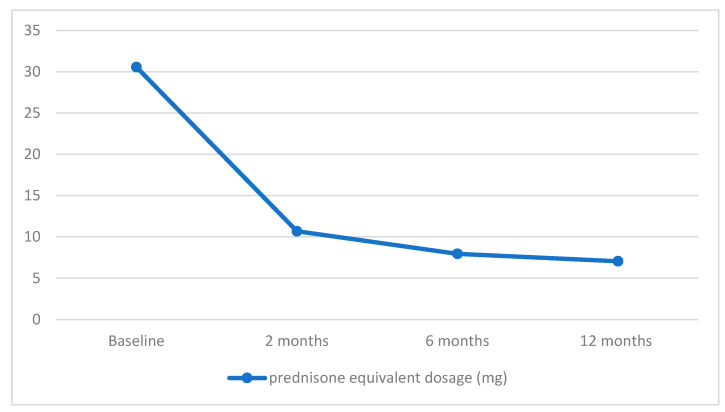
Decrease in mean prednisone equivalent dosage during rituximab (RTX) therapy in AIBD.

The data on patients’ response to RTX therapy during the 1-year follow-up period are depicted in Figure 1.

We calculated the levels of DSG-1 and DSG-3 exclusively for the pemphigus patient group, consisting of 10 individuals.

The mean initial titers of autoantibodies directed against DSG-1 and DSG-3 were 2.61 ± 2.62 and 3.73 ± 2.59, respectively.

Two months post the last course of RTX, the values decreased to 1.39 for DSG-1 and 2.69 for DSG-3. Subsequently, at the 6-month mark, the antibody values for DSG-1 further diminished to 0.7. However, over the course of a year of observation, they exhibited a slight increase to 1.2. By contrast, the concentration of antibodies for DSG-3 demonstrated an increase compared to the levels observed 2 months after RTX administration, rising to 3.19 after 6 months and further to 3.54 after 12 months.

Table 2 displays the titers of desmoglein levels before and after RTX treatment.

As an overview, relative to the initial antibody concentrations for DSG-1 and DSG-3, there were reductions of 43.29%, 75.86%, and 54.02% for DSG-1 and 27.88%, 14.48%, and 5.09% for DSG-3 at the intervals of 2, 6, and 12 months following the last administration of RTX.

**Figure 2:**
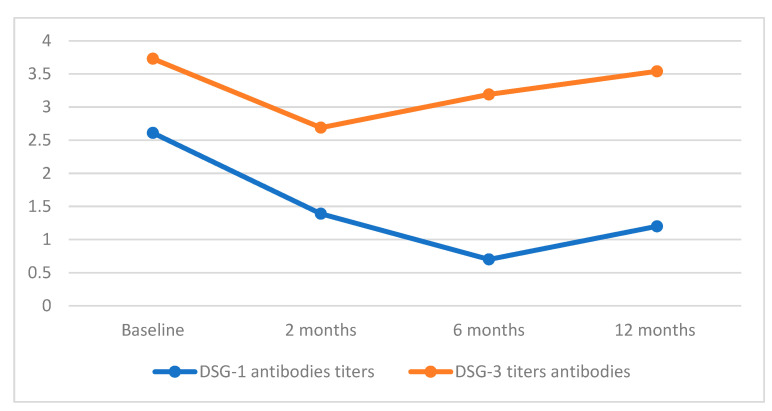
Desmoglein concentrations post one-year of RTX treatment.

Figure 2 illustrates the data pertaining to changes in the titers of DSG-1 and DSG-3 following one year of follow-up after RTX therapy.

Two months post-treatment completion, 18.18% of patients experienced disease recurrence, and this rate persisted for up to one year. Throughout the six-month observation period, none of the patients achieved CRNT. However, after 12 months, one individual (9.09%) remained in complete remission without therapy. CRMT and PRMT were observed in 63.64% and 18.18% of patients, respectively, at the two-month mark. In the 12-month follow-up, the percentage of patients achieving CRMT decreased to 54.55%, while the proportion of those with PRMT remained unchanged.

**Figure 3:**
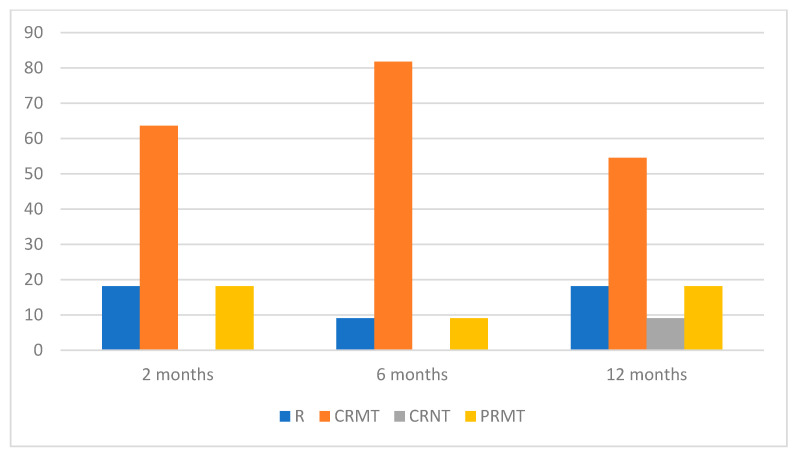
Percentage distribution of treatment effects during RTX therapy in AIBD.

The data for patients’ response to RTX therapy during the 1-year follow-up are shown in Figure 3.

In our study, we enrolled patients exhibiting severe forms of AIBD, either unresponsive to diverse therapeutic interventions or necessitating dose reduction due to severe side effects. All subjects underwent RTX administration within a Ministry of Health-sponsored government program. Eligibility for inclusion in the program necessitated the fulfillment of at least one of the following criteria: a confirmed diagnosis of paraneoplastic pemphigus; contraindications to systemic corticosteroid therapy; dependence on GCS for pemphigus management; and refractoriness to GCS treatment. It is notable that the abovementioned criteria differ significantly from the recommendations outlined in current guidelines, specifically deviating from the immediate initiation of RTX upon the diagnosis of pemphigus [7].

**Figure 4:**
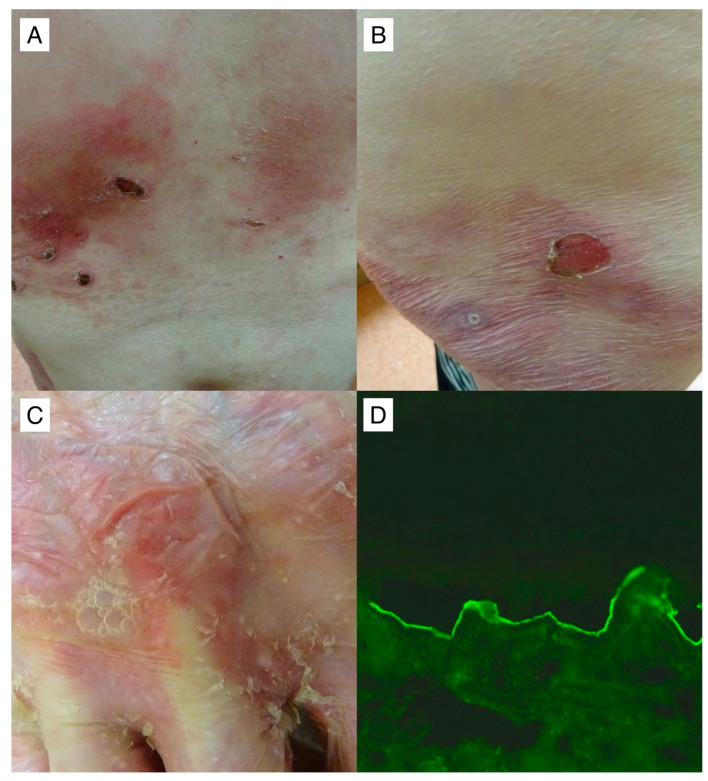
A noteworthy 62-year-old patient with recalcitrant mechanobullous EBA (#11) in whom the value of IgG antibodies to type VII collagen with multiplex ELISA 3 months post two RTX infusions was highly elevated 4.51 (cut-off value = 1). Despite the RTX treatment regimen, the patient exhibited no clinical improvement. Erosions on an erythematous background are shown, some of which, covered with crusts, were seen on the trunk (**A**) and thigh (**B**). A flaccid blister with air bubbles and exfoliation in its vicinity on the dorsal surface of the hand was visible (**C**). IgG4 antibodies (**D**), but not IgG antibodies, reacting with the dermal side of salt–split primate skin at a titer above 1:160 were detected with IIF mosaic (Euroimmun, Germany with our own modification) performed simultaneously with multiplex ELISA and visualized with a blue light-emitting diode technology-operated microscopy with an original objective magnification of ×40.

Noteworthy is the singular case of a patient with EBA (Figure 4), wherein disease progression persisted despite repeated attempts at treatment utilizing various immunosuppressants. As this patient did not meet the eligibility criteria for RTX treatment within the government program, we pursued an application with a charitable foundation, which unfortunately yielded a negative response. Subsequently, the hospital administration forwarded our request for drug financing to the Minister of Health. However, a delay of four months ensued while awaiting the assessment from another government entity. While the decision eventually turned out to be positive, it is regrettable that the protracted approval process led to the continued advancement of the disease. Three months post-RTX administration, the disease remained active, with the patient exhibiting a therapeutic response to high doses of intravenous methylprednisolone.

Adverse events were observed in four patients, constituting 36.16% of the study cohort, and were characterized as mild. Specifically, two patients (designated as #4 and #8) manifested upper respiratory tract infections within a few weeks following RTX administration. Notably, these infections were of a mild nature, did not necessitate hospitalization, and resolved spontaneously.

In the case of patient #6, a notable event occurred during the initial infusion of RTX, wherein a decrease in blood pressure transpired 30 min into the infusion. The infusion was promptly interrupted, and after an hour, it was resumed at a slower rate, with subsequent observations indicating no further declines in blood pressure. Importantly, no analogous event was recorded during the subsequent administration of RTX.

Patient #2 experienced a distinct adverse event a few hours following the second RTX infusion, reporting severe nausea. To address this, antiemetics were administered, resulting in the prompt alleviation of symptoms.

## 4. Discussion

In our study group, following the introduction of rituximab, a substantial decrease in GCS usage and a concurrent reduction in disease activity were noted over the course of one year. The manageable side effects of rituximab did not result in any serious sequelae for any participant. Despite the extended time from disease diagnosis to RTX initiation and the inclusion of non-responsive patients, the majority experienced benefits from RTX treatment. 

RTX’s efficacy in refractory pemphigus cases is well-established in numerous studies [11,12]. In our cohort of severe AIBD patients, 81.82% achieved partial or complete remission within one year post-RTX administration with minimal or no additional therapy, underscoring the efficacy of this treatment modality. Balghi et al. reported that patients treated with RTX within six months of diagnosis had a higher likelihood of achieving complete remission (CR), irrespective of the time taken to reach remission. In our study group, all individuals received treatment more than one year from diagnosis, yet 7 out of 11 attained CR. This disparity from Balghi et al.’s findings suggests the presence of additional factors influencing responsiveness to RTX treatment in pemphigus patients [13].

Moreover, our study group exhibited a notable 76.93% reduction in the initial GCS dose one year after the initiation of RTX, underscoring its effectiveness in patients resistant to standard treatment methods within our cohort. Joly et al. initially and, later, Chen et al. both reported increased efficacy in achieving remission and reducing the total GCS dose in pemphigus patients treated with a combination of RTX and GCS compared to GCS alone [14,15]. Following the introduction of RTX, a significant reduction in the GCS dose and attainment of clinical remission were observed for the majority of participants in our research, which is consistent with the findings in the abovementioned studies. In a study conducted by Werth et al., pemphigus vulgaris patients treated with RTX experienced a more substantial reduction in the GCS dose compared to the group treated with mycophenolate mofetil. This indicates that promptly integrating RTX into AIBD therapy, irrespective of disease duration, aids in alleviating the side effects associated with GCS treatment [16].

Amber et al. demonstrated a substantial and diverse array of autoantibodies in pemphigus, encompassing several hundred antibodies. Some identified antibodies included desmocollins, anti-mitochondrial antibodies, thyroid peroxide antibodies, plakophilin 3, e-cadherins, and plakoglobin [17]. Another potential factor contributing to non-response to RTX treatment was the detection of human anti-chimeric antibodies (HACA) targeting the murine components of rituximab [18]. Lunardon and Payne reported a case of a woman with pemphigus vulgaris diagnosed with elevated HACA antibodies exhibiting no response to RTX treatment [19]. Furthermore, In AIBD patients, the presence of diffuse ectopic lymphoid-like structures in the skin and mucous membranes may render B cells insensitive to RTX therapy, posing challenges in achieving remission [20]. Our team documented a case of PV in a patient with a persistent single lesion on the head for over 2 years, sustained by an ectopic lymphoid-like structure. The combination of intralesional therapy and systemic GCS with RTX led to the reversal of these changes, underscoring the necessity of combining RTX with other therapeutic modalities in similar patients. The multitude of factors discussed above complicates the evaluation of RTX treatment response in AIBD patients, underscoring the need for additional research [21].

Our study group exhibited a noteworthy reduction in DSG-1 levels following a 12-month period post-RTX, contrasting with a decrease in DSG-3 levels after 2 months, which, however, showed a marginal decline below the baseline levels after 12 months. The observed substantial improvement in patients’ post-treatment suggests a potential correlation between disease activity and DSG-1 levels, as opposed to DSG-3. This finding aligns with the existing literature; however, more research is needed in this area [22,23]. Patients #9 and #10, experiencing a relapse after 12 months, consistently exhibited elevated DSG-3 levels during the entire follow-up period, suggesting a potential lack of response to rituximab treatment. 

While the short-term efficacy of RTX is well-established in numerous studies, its long-term effectiveness remains a subject of debate [24,25]. Its mechanism involves CD20 binding, resulting in B-cell depletion through diverse pathway complement-triggered cell destruction, antibody-dependent cellular killing, and the initiation of apoptosis [26].

The response to RTX in pemphigus patients is influenced not only by the total B cell count but also by autoreactive DSG-specific B cells and T follicular helper cells [27,28]. Colliou et al. demonstrated that RTX increases naïve CD19 + CD27 − cells while delaying the creation of CD19 + CD27 + B memory cells. This inhibition of antibody class transformation from IgM to IgG suppresses anti-desmoglein IgG antibody formation, contributing to long-term clinical remission in pemphigus patients [28]. Maho-Vaillant M et al. affirmed that the prolonged elimination of anti-DSG-1 and anti-DSG3 IgG+ antibodies by RTX is associated with extended remission time. The authors observed that the only patient from their cohort with active disease exhibited elevated serum titers of anti-DSG-3 IgG+ antibodies, aligning with the heightened levels of B cells producing these antibodies [29]. This finding supports the notion that consistently elevated DSG-3 levels in our patients may be linked to disease relapse. Future considerations should include detailed assessments of IgG+ antibodies and B cell levels to precisely determine the optimal discontinuation point for RTX and the potential need for maintenance therapy.

EBA is a rare dermatosis with often unsatisfactory treatment outcomes. Evidence suggests that RTX holds promise as an efficacious therapeutic intervention, substantiated by documented successes in a limited number of patient cases within the existing literature [30,31]. A patient enrolled in our study cohort, diagnosed with EBA, exhibited initial resistance to RTX intervention. Subsequent to the administration of escalated GCS doses, remission was attained. In the course of long-term evaluation, a judicious tapering of GCS dosages ensued, culminating in the achievement of CRMT. The inefficacy of RTX in treating EBA may be attributed to the intricate nature of this disease’s patomechanism. This complexity encompasses the production of autoantibodies against COL-7, which is reliant on T cells. Additionally, the presence of certain autoantibodies within the IgA subclass, originating from B-cell precursors, could render them more resistant to the effects of RTX [32,33]. Dasdar et al. demonstrated, based on a study involving 15 EBA patients, that a less favorable response to RTX was associated with the duration between the diagnosis of the disease and the commencement of RTX treatment, which is consistent with our patient’s observation [34].

While RTX is generally considered safe, it is crucial to acknowledge that it is typically administered to patients with diverse medical conditions [35]. A common side effect of RTX is the increased risk of infections caused by persistent B-cell depletion [36,37]. Moreover, patients with AIBD are often additionally treated with GCS, which increases the risk of severe infections [38]. Therefore, a meticulous evaluation is imperative to identify any contraindications to treatment, particularly active infections. Two patients from our group experienced self-resolving mild infections. However, in patients after RTX treatment, ongoing vigilance during follow-up visits is essential to promptly identify and address any symptoms of active infections so as to mitigate the risk of complications. 

Hypotension, a plausible infusion-related reaction, commonly emerges within two hours of drug administration. Effective prevention involves adjusting the infusion rate, as exemplified by our patient’s case [39,40]. Patients undergoing RTX treatment may experience gastrointestinal issues, including nausea, diarrhea, and vomiting. While the exact cause is uncertain, symptomatic treatment proved effective in addressing these complaints in a patient in our study [41].

This research encountered specific constraints, including a restricted sample size. The scarcity of AIBD, coupled with the elevated expenses and numerous administrative hurdles associated with RTX therapy, contributed to the limited participation pool. Moreover, the homogeneity of our group was compromised by the inclusion of one patient diagnosed with EBA. Furthermore, relying on the retrospective analysis of visual documentation and depending on patient files for data interpretation can introduce potential vulnerabilities to our study. While the 1-year follow-up period post-RTX administration is substantial, a protracted follow-up duration could enhance the precision of monitoring antibody levels and gauging the response to RTX treatment.

## 5. Conclusions

In summary, RTX stands as the primary treatment modality for pemphigus, with potential efficacy extending to other AIBDs. In instances where the initial use of RTX is impractical for various reasons, our research supports its incorporation as a secondary intervention for patients unresponsive to alternative treatments.

While some patients may exhibit non-responsiveness to treatment, and many necessitate supportive therapy, particularly in the form of low-dose oral GCS, the commendable safety profile of RTX, coupled with its potential for significant GCS dose reduction, underscores its merit as a viable therapeutic option.

Despite RTX’s long-standing availability, challenges persist in terms of treatment accessibility and cost. Furthermore, a comprehensive exploration through additional studies is imperative to determine the optimal threshold of disease activity warranting treatment solely with GCS, as opposed to RTX. Additionally, ongoing research is crucial for establishing whether anti-DSG antibodies can serve as effective disease monitors and whether the initiation of RTX should be contingent on initial antibody levels. 

Further research is crucial to unravel the cellular-level mechanisms affecting RTX response, particularly in patients lacking sustained remission. Investigation into assessing total B cell levels and the IgG desmoglein fraction is warranted, offering insights into the specific risk of disease recurrence and guiding potential maintenance therapy strategies.

## Figures and Tables

**Table 1:** Patients’ demographics, treatment methods, and treatment outcomes.

Patient	Gender/Age (Years)	Disease Type	Duration of Disease Prior to RTX Therapy (Years)	Prior Treatments	Supplementary Medications	Adverse Reactions	Management of Disease Activity after		
							2 Months	6 Months	12 Months
1	F/62	PV	7	OGCS, IGCS, DP, CPH, PPH, IVIG	None	None	CRMT	CRMT	CRMT
2	M/68	PF	10	OGCS	None	Nausea	CRMT	CRMT	PRMT
3	F/27	PV	3.5	OGCS, IGCS, RTX (2 × 1 g)	DP 25 mg/day	None	CRMT	CRMT	CRMT
4	F/38	PV	1	OGCS, IGCS	None	Infection	PRMT	R	PRMT
5	M/68	PV	3.5	OGCS, CPH	None	None	R	CRMT	CRMT
6	F/69	PV	10	OGCS, DP, PPH	None	Hypotension	CRMT	CRMT	CRMT
7	F/40	PF	1	OGCS, DP	DP 25 mg/day	None	CRMT	CRMT	CRMT
8	F/46	PV	12	OGCS, IGCS, DP, CPH	None	Infection	CRMT	CRMT	CRNT
9	F/46	PV	2	OGCS, IGCS, CPH, IVIG (1 infusion)	None	None	PRMT	CRMT	R
10	M/54	PV	2.5	OGCS, DP	DP 50 mg/day	None	CRMT	PRMT	R
11	F/62	EBA	3.5	OCGS, PPH, DP, AZA, MTX, CPH	3xIGCS 1 g	None	R	CRMT	CRMT

PF, pemphigus foliaceus; PV, pemphigus vulgaris; EBA, epidermolysis bullosa acquisita; CPH, cyclophosphamide; IGCS, intravenous glucocorticosteroids; IVIG, intravenous immunoglobulin; PPH, plasmapheresis; AZA, azathioprine; RTX, rituximab; OGCS, oral glucocorticosteroids; MTX, methotrexate; DP, dapsone; CRNT, complete remission off (no) therapy; CRMT, complete remission on minimal therapy; PRMT, partial remission on minimal therapy; R, relapse.

**Table 2:** DSG-1 and DSG-3 levels before and after RTX infusions.

Patient	DSG-1 Level				DSG-3 Level			
	Before RTX	2 Months	6 Months	12 Months	Before RTX	2 Months	6 Months	12 Months
1	5.87	4.42	0.25	0.22	0.63	0.25	3.01	3.35
2	6.25	3.67	2.21	3.06	0.15	0	0.14	0.26
3	1.03	0.27	0.22	0.25	4.42	2.17	2.46	3.45
4	0.85	0.55	1.12	1.53	7.03	4.44	6.44	6.13
5	0.72	0.67	0.15	0.19	6.22	5.46	5.74	5.65
6	0.63	0.38	0.73	0.77	3.48	2.92	2.88	2.6
7	1.86	0.84	0.64	0.62	0.15	0.12	0.11	0.1
8	0.44	0.33	0.12	0.2	2.8	1.7	1.03	0.92
9	7.47	2.54	1.28	3.96	6.86	6.7	5.62	6.62
10	0.94	0.23	0.32	1.33	5.57	3.14	4.56	6.31

## Data Availability

The datasets generated and/or analyzed in the current study are not publicly available due to data privacy but are available from the corresponding author on reasonable request.
